# Pain-related psychological cognitions and behaviours associated with sick leave due to neck pain: findings from the Nurses and Midwives e-Cohort Study

**DOI:** 10.1186/1472-6955-13-5

**Published:** 2014-02-24

**Authors:** Philip J Schluter, Anna P Dawson, Catherine Turner

**Affiliations:** 1 School of Health Sciences, University of Canterbury, Private Bag 4800, Christchurch, CHCH 8140, New Zealand; 2 School of Nursing and Midwifery, University of Queensland, Brisbane, QLD 4072, Australia; 3 Sansom Institute for Health Research, University of South Australia, Adelaide, SA 5000, Australia

**Keywords:** Neck pain, Sick leave, Psychosocial factors, Nursing and midwifery, Epidemiology

## Abstract

**Background:**

Sick leave due to neck pain (NP-SL) is costly and negatively impacts the productivity of the nursing and midwifery workforce. Identification of modifiable risk indicators is necessary to inform preventive efforts. This study aimed to investigate the role of pain-related psychological features (pain catastrophizing, fear of movement, and pain coping) in NP-SL alongside other potential risk indicators.

**Methods:**

A cross-sectional analysis of a large cohort study of Australian and New Zealand nurses and midwives, established between 1st April 2006 to 30th March 2008, was undertaken. Recruitment procedures adopted within each Nursing Council jurisdiction were governed by the individual regulatory authorities and their willingness to engage with the study. Invitations directed potential participants to a purpose-built internet-based survey, where study information was provided and consent requested. Once consent was obtained, a range of standardized tools combined into one comprehensive electronic questionnaire was elicited. Exposure variables assessed included pain characteristics and a broad range of psychological, psychosocial, occupational, general health and demographic factors. Two-way interactions between age and gender and candidate exposures were also assessed. Binary logistic regression was performed using manual backward stepwise elimination of non-significant terms.

**Results:**

The cohort included 4,903 currently working nurses or midwives aged 18–65 years. Of these, 2,481 (50.6%) reported neck pain in the preceding 12 months. Our sample comprised of 1,854 working nurses and midwives with neck pain in the preceding year who supplied sick leave data. Of these, 343 (18.5%) reported taking sick leave in the preceding year due to their neck pain. The final most parsimonious multivariable model demonstrated neck pain severity (adjusted odds ratio, [aOR] = 1.59), passive pain coping (aOR = 1.08) and fear of movement (aOR = 1.06) increased the likelihood of NP-SL in the previous year. Interactions between demographic and general health factors exhibited both protective and risk relationships with NP-SL, and there was no association between pain catastrophizing and NP-SL.

**Conclusions:**

Findings demonstrate that sick leave due to neck pain was associated with pain severity, fear of movement and passive pain coping. In addition, there were complex interactions found between demographic and general health factors. These features represent potentially modifiable targets for preventive programs.

## Background

Neck pain (NP) appears to be more prominent in the general population than previously considered [1]. It affects up to 71% of individuals during their lifetime [2], and general population studies indicate that between 30-50% of individuals will experience NP in any 12-month period (range: 12.1%-71.5%) [3]. Furthermore, it is commonly recurrent, with over 50% of workers with NP reporting symptoms a year later [4]. Economic analyses reveal that NP can place a considerable annual financial burden on society [3,5,6]. In the Netherlands, sick leave due to neck pain (NP-SL) was reported to comprise over a quarter of the total NP financial burden, and costed an estimated US$185.4 million from 1.4 million days of lost productivity associated with worker absence [5].

Given the societal, workforce and personal implications of NP-SL, the factors associated with NP-SL must be understood in order to inform preventive efforts. Exposures examined to date have predominated around pain characteristics and occupational, demographic and psychosocial factors with inconsistent results [7-13]. In addition, there has been only one study of factors associated with NP-SL as an exclusive outcome [13]. This analysis showed that work-related neck flexion and neck rotation, low decision authority and medium skill discretion were significantly related to NP-SL in industrial workers [13]. All other studies have examined work absenteeism due to pain in composite regions that included the neck in combination with the shoulder, upper extremity and/or upper and lower back [7-12]. Given that factors related to composite outcomes may differ to those related to NP-SL in isolation, analyses of sick leave due to pain in the neck region alone are needed to clarify exposure relationships.

The relationship between NP-SL and pain-related psychological features has never been investigated. Theoretical models posit that mal-adaptive pain-related cognitions and behaviours contribute to development of chronic pain [14,15]. Specifically, fear-avoidance theory asserts that catastrophizing about pain leads to pain-related fear (e.g. fear of movement) and avoidance/escape behaviours (e.g. passive pain coping) that lead to disability, disuse, and further pain [14,15]. Staying home from work is a potential proxy of avoidant behaviour, hence mal-adaptive pain-related features may play a role in explaining NP-SL. The demonstrated relationship between pain-related psychological features and sick leave due to low back pain [16,17] suggests these factors may be similarly related to NP-SL. Given that psychological features may be modifiable, and therefore ideal targets for preventive efforts, their role in NP-SL must be defined.

Utilizing a large occupational cohort of Australian and New Zealand nurses and midwives, the aim of this study was to determine the role of pain-related psychological factors (pain catastrophizing, fear of movement and pain coping) and other psychosocial, occupational, demographic and pain characteristics in explaining NP-SL. Under the Australian National Employment Standards, full-time employees are entitled to 10 days’ paid personal leave (for sick and paid carer’s leave) per year. Part-time employees receive a pro rata entitlement to sick leave based on the number of hours they work. In New Zealand there is a minimum provision of five days’ paid sick leave a year after the first six months of continuous employment and an additional five days’ sick leave after each subsequent 12-month period. Leave accumulates from year to year in both countries. The nursing population was targeted to answer our research questions since NP negatively impacts this profession. A population study of multiple occupational groups in Britain found that nurses reported the highest annual prevalence (15%) for NP that prevented normal activities [18]. Further, workforce attrition studies show than nurses with neck/shoulder pain are 1.5 times more likely to leave nursing work [19]. Hence, an understanding of the factors associated with NP-SL can inform professional policy and preventive programs to maximise the viable nursing workforce.

## Methods

### Participants, study design, and procedure

The sample comprised Australian and New Zealand nurses and midwives recruited as part of the Nurses and Midwives e-Cohort Study (NMeS). The NMeS is a longitudinal study that investigates workforce and health outcomes in nurses and midwives from Australia, New Zealand and the United Kingdom (UK) using innovative online (http://www.e-cohort.net) data collection methods [20]. Recruitment strategies, response rates and characteristics of the NMeS sample have previously been described in detail [21,22]. In brief, invitations for participation varied between the varying State-based and national Nursing and Midwifery Council jurisdictions, and included: flyers within licence renewal packages (Queensland, Tasmania, New Zealand), postcards mailed to home addresses (Australian Capital Territory, New South Wales, Western Australia), and mass advertisement and/or articles available and circulated to Council members (Northern Territory, South Australia, Victoria). Invitations directed potential participants to a purpose-built internet-based survey (see: http://www.e-cohort.net). Upon entering the study web-site, detailed information was provided and consent requested. Once received, participants established their personal profile and completed the registration page which requested baseline demographic, contact and work-related details. Participants were then directed to the baseline survey, which contained various standardized instruments around the Work/Life Balance and Staying Healthy study themes and took approximately 40 minutes to complete. Participants with incomplete surveys were emailed reminders approximately two weeks, four weeks and six weeks following initial registration within the NMeS. Recruitment, registration and the baseline survey was open from 1 April 2006 to 31 March 2008. Follow-up surveys have taken place approximately bi-annually: the first opened on 30 August 2008 and closed 26 September 2009, and the second opened on 25 May 2010 and closed on the 31 May 2011. The cross-sectional data used in this analysis was taken from the NMeS baseline survey collected in 2006–2007. Participants included in this analysis were aged 18–65 years, currently employed in nursing or midwifery work, had professional registration in Australia or New Zealand and reported NP in the preceding 12 month period. As UK nurses and midwives were recruited using opportunistic strategies, as opposed to the targeted approach used in Australia and New Zealand, they were omitted from this study to minimize responder bias. Overall, 7,812 (2.3%) eligible nurses and midwives participated from a total pool of 334,400. However, age, gender, occupational and health profiles were similar between countries and to national workforce figures [21,22].

Ethical approval for the NMeS was granted clearance by the University of Queensland’s Behavioural and Social Science Ethics Review Committee (No. 2005000696) and the Massey University Human Ethics Committee (Wellington: No. 05/71), and all participants provided informed consent.

### Outcome measure

The outcome for all analyses was whether NP-SL was taken in the previous year, using a binary (Yes/No) variable. A question from the General Standardised Nordic Questionnaire was used to determine 12-month NP prevalence [23]. Neck trouble was defined as ‘ache, pain or discomfort in the area extending from the base of the skull to the top of the trunk and shoulder region’.

### Exposure measures

Pain, psychosocial, demographic, occupational, and general health factors were all investigated.

#### Pain characteristics

*Neck pain severity* (rating of worst pain on an 11-point scale, 0–10) was measured with an item from the Graded Chronic Pain Scale adapted to inquire about the preceding 12 month interval [24].

#### Psychosocial factors

Four psychosocial domains identified by Linton [25] that are relevant to neck and back pain were assessed: cognitive, emotional, social and behavioural.

(i) *Cognitive: Pain catastrophizing* is an exaggerated negative orientation to pain [26] that includes “performing cognitive activities that exacerbate the fearful aspects of the pain experience” [27]. We used the reliable and valid Pain Catastrophizing Scale to assess this construct, using its three subscales rumination (range 0–16 points), magnification (range 0–12 points), and helplessness (range 0–24 points) [26,28]. *Fear of movement* was assessed using the 11-item version of the Tampa Scale of Kinesiophobia (TSK-11) (range 11–44 points) that has demonstrated reliability and validity in patients with chronic low back pain [29]. The scale assesses the construct kinesiophobia, which is defined as “an excessive, irrational and debilitating fear of physical movement and activity resulting from a feeling of vulnerability to painful injury or re-injury” [29]. Respondents were asked to answer TSK-11 questions specific to their NP experience. We also asked participants whether they believed nursing work caused or exacerbated their NP, and quantified this belief with a binary (Yes/No) variable we named *Belief that nursing work caused/exacerbated NP*.

(ii) *Emotional*: The 10-item version of the Centre for Epidemiologic Studies Depression Scale (CESD-10) was used to assess depression [30]. A *current depressive symptom*s binary (Yes/No) variable was generated using the cut-off score of ≥10 [30]. Prior *diagnosis of anxiety and diagnosis of depression* by a medical professional were assessed with binary (Yes/No) variables. A *general mental health* status measure was included using the Mental Health Component Score from the SF-36 (Version 2) Health Survey, standardised to 1998 United States general population normative values [31]. Higher scores on this scale reflect better health.

(iii) *Social*: The Job Content Questionnaire (JCQ) (Version 1.1) was used to assess the following work-related constructs: *job control* (the sum of Skill Discretion + Decision Authority subscales), *social support* (the sum of Co-Worker Support + Supervisor Support subscales) and *psychological demands*[32]. The Effort-Reward Imbalance Questionnaire (ERIQ) was used to assess *effort-reward imbalance* (ERI) and *over-commitment* (OC) [33]. Possibly due to challenges of electronic administration of the ERIQ [22], 69% of ERI data was missing and consequently ERI was not included in analyses. OC measures the inability to withdraw from work obligations [33], and is categorized into high (upper tertile of the sample distribution) and low (lower two tertiles) [34].

(iv) *Behavioural*: *pain coping* was assessed with the 11-item abbreviated version of the Vanderbilt Pain Management Inventory (VPMI) [35,36] that has established internal consistency and factorial validity in individuals with spinal pain [37]. The instrument generates two subscales: *active coping* and *passive coping*. The active coping subscale assesses such strategies as functioning in spite of pain or distracting oneself from pain (score 5–25 points) and the passive coping subscale assesses behaviours such as depending on others for pain management assistance and allowing pain to adversely impact usual activities (score 6–30 points). As for the TSK-11, respondents were requested to answer VPMI questions specific to their NP experience.

#### Demographic factors

The demographic factors included in the model were *gender*, *marital status*, *country* and *age*, with age standardised to years at 30th November 2006.

#### Occupational factors

A categorical variable named *job classification* was used to quantify nursing and midwifery work into five groups that delineate qualifications and clinical/non-clinical work: (i) registered nurses and midwives; (ii) enrolled nurses and nurse assistants; (iii) specialist registered nurses and midwives (e.g. nurse practitioners, clinical educators); (iv) administrators, managers and policy makers; and, (v) academics and university educators. *Current shift work* was assessed with a binary variable (Yes/No). *Manual handling task frequency* was assessed with an 8-item specially-developed instrument (total score 0–24 points). The instrument inquires about frequency (assessed with a Likert scale 0, 1–4, 5–10, 11+ that is correspondingly scored 0–3) of manual handling activities such as assisting patients to transfer from sit to stand and pushing/pulling lifting equipment. *General physical demands* was assessed with the JCQ subscale of the same name that includes two questions relating to physical effort and rapid and continuous physical activity.

#### General health factors

A range of general health factors that could potentially have a confounding influence were included in analyses. *Current pregnancy*, *hours of sleep* and *smoking status* variables were included, and the Physical Health Component Score of the SF-36v2, standardised to 1998 US population normative data [31], was used as a measure of *general physical health*. Finally, a range of diagnosed conditions (*asthma*, *pneumonia*, *osteoarthritis*, *rheumatoid arthritis*, *chronic obstructive pulmonary disease*, *multiple sclerosis*, *Parkinson’s disease*, *stroke*, *breast cancer*, *congestive heart failure*, *high blood pressure*, *lung cancer*) were considered in analyses using binary (Yes/No) variables.

### Test-retest reliability

Measures used within the NMeS baseline survey were sourced from existing instruments with established psychometric properties when available. When questionnaires were unavailable or inappropriate, questions were constructed or adapted and assessed for test-retest reliability in a sub-study of 32 nurses and midwives. These nurses and midwives re-completed the baseline NMeS survey on average 8.8 ± 2.5 days (range 5–16) after their first completion. The reliability of categorical data was assessed with proportion of observed agreement (*p*_o_) and the kappa statistic (κ). Specifically, the maximum kappa obtainable (κ_max_) was calculated, and the proportion of κ_max_ achieved (κ/κ_max_) was used to represent test-retest reliability [38]. The reliability of continuous variables was assessed with the intra-class correlation coefficient using two-way models with random effects and single measures (ICC_[2, 1]_) [39]. The reliability of variables assessed in this way was acceptable: annual sick leave due to neck pain, *p*_o_ = 0.81, κ/κ_max_ = 0.74; belief that nursing work caused/exacerbated NP, *p*_o_ = 0.82, κ/κ_max_ = 0.39; pain severity ICC_[2, 1]_ = 0.73 (95% confidence interval [CI]: 0.42, 0.89); and manual handling task frequency ICC_[2, 1]_ = 0.98 (95% CI: 0.95, 0.99).

### Statistical analysis

The characterisation of some candidate exposure variables could be expressed in multiple forms (e.g. continuous, categorical, dichotomous, as well as individual versus composite variables). Selection between these characterisations were made in bivariable logistic regression models using the Bayesian Information Criterion (BIC) [40]. The BIC penalises for model complexity and rewards for goodness-of-fit; with the preferred model balancing these competing demands and yielding the lowest BIC value. On this basis, for example, the pain catastrophising subscales were included in multivariable modeling over the total pain catastrophising score, and the composite JCQ variables (i.e. job support and job control) were selected rather than individual variables. Crude relationships between the outcome and all exposure variables were then assessed using bivariable logistic regression. Since confounding can lead exposures with modest bivariable associations to have significant relationships in multivariable models [41], a conservative threshold for inclusion in multivariable analysis was set at p < 0.40. Next, all exposure variables yielding a bivariable association with p < 0.40 were included in a multivariable main effects logistic regression model. Non-significant exposure variables were then sequentially omitted from the model if they failed to reach then p < 0.40 criterion. This process determined the most parsimonious main effects model. At this stage, age and gender were retained within the statistical model, regardless of significance. Next, all two-factor interaction terms between age and gender and all remaining exposure variables were entered into the model. Stepwise elimination of non-significant interaction terms was then undertaken using a p < 0.05 criterion. Non-significant main effect variables that did not form part of a significant interaction term were then also eliminated, in a stepwise fashion, using the p < 0.05 until the final model was ascertained. The goodness-of-fit of the final model was assessed with the Hosmer-Lemeshow test [42]. Multicollinearity between regressors was evaluated by tolerance and condition index estimates derived from linear regression models. All analyses were undertaken using Stata version 12.0 (StataCorp, College Station, Texas, USA).

## Results

### Sample characteristics

The NMeS recorded 10,721 participant registrations, which equated to 10,112 unique registrants following removal of duplicates (some individuals registered on more than one occasion). Of 8,651 individuals who went on to complete the online survey instrument, 7,812 were professionally registered in Australia or New Zealand and 4,903 were aged 18–65 years and were currently working in a nursing or midwifery role. Of these, 50.6% of these individuals reported NP in the preceding 12 months. Our study sample for the present analysis is comprised of 1,854 individuals with NP in the preceding year that provided data regarding sick leave (i.e. NP-SL) during that period. Compared to those who responded to the NP-SL question, non-responders were older (45.0 years verses 43.9 years, p = 0.004), more likely to be female (95.4% versus 92.9%, p = 0.03), and more likely to be Australian (82.4% versus 88.8%, p < 0.001), but no different in marital status (p = 0.35) or job classification (p = 0.08) profiles. The differential completion of the sick leave response question by eligible participants who reported NP in the preceding 12 months may introduce bias in pursuant prevalence estimates and reported associations.

### Sick leave due to neck pain characteristics

Overall, 343 (18.5%) nurses and midwives our study sample reported taking sick leave in the preceding year due to their NP, while 1,511 (81.5%) took no leave due to this pain. Table 1 presents the demographic, occupational and general health characteristics of the sample partitioned by the dichotomous sick leave status, together with the bivariable associations between these factors and NP-SL. Based on the p < 0.40 exclusion criterion, variables measuring co-morbid low back pain, pregnancy, and smoking status were not considered within the multivariable model development. Variables measuring age and gender were retained at this stage.

**Table 1:** Demographic, occupational and general health characteristics of Australian and New Zealand nurses and midwives with neck pain in the preceding 12-months by whether they took no sick leave (n = 1511) or sick leave (n = 343), together with the bivariable associations between these factors and neck pain related sick leave (NP-SL)

	**No sick leave taken**		**Sick leave taken**		**OR**	**(95% CI)**	**p-value**
**Demographic factors**							
*Age in years*, mean (SD)	44.3	(9.3)	42.1	(9.8)	0.98	(0.96, 0.99)	<0.001
*Gender*, n (%)							0.52
Female	1407	(93.1)	316	(92.1)	1.00	(reference)	
Male	104	(6.9)	27	(7.9)	1.16	(0.74, 1.80)	
*Marital status*, n (%)							0.37
Never married	239	(16.1)	64	(18.9)	1.00	(reference)	
Married/de facto	1049	(70.4)	235	(69.3)	0.84	(0.61, 1.14)	
Divorced/separated/widowed	201	(13.5)	40	(11.8)	0.74	(0.48, 1.15)	
*Country*, n (%)							0.01
New Zealand	283	(18.7)	44	(12.8)	1.00	(reference)	
Australia	1228	(81.3)	299	(87.2)	1.57	(1.11, 2.20)	
**Occupational factors**							
*Job classification*, n (%)							0.36
Registered nurse/midwife	700	(67.0)	143	(60.6)	1.00	(reference)	
Enrolled nurse/nurse assistant	85	(8.1)	27	(11.4)	1.56	(0.97, 2.49)	
Specialist registered nurse/midwife	125	(12.0)	32	(13.6)	1.25	(0.82, 1.92)	
Administrator/manager	98	(9.4)	25	(10.6)	1.25	(0.78, 2.01)	
Academic/university lecturer	36	(3.4)	9	(3.8)	1.22	(0.58, 2.60)	
*Current shift work*, n (%)*	825	(54.8)	214	(62.8)	1.39	(1.09, 1.77)	0.01
*Manual handling frequency*, mean (SD)	5.7	(6.0)	6.9	(7.0)	1.03	(1.01, 1.05)	0.002
*Physical demands*, mean (SD)	4.9	(1.6)	5.1	(1.6)	1.08	(1.00, 1.16)	0.04
**General health factors**							
*General physical health*, mean (SD)	50.9	(7.2)	47.5	(8.3)	0.95	(0.93, 0.96)	<0.001
*Co-morbid low back pain*, n (%)*	1061	(71.5)	237	(70.4)	1.06	(0.82, 1.37)	0.68
*Pregnant*, n (%)*	35	(2.4)	6	(1.8)	0.76	(0.32, 1.83)	0.54
*Asthma*, n (%)*	327	(22.9)	95	(29.5)	1.41	(1.08, 1.85)	0.01
*Emphysema/chronic bronchitis*, n (%)*	30	(2.1)	3	(0.9)	0.44	(0.13, 1.50)	0.18
*Pneumonia*, n (%)*	141	(9.8)	25	(7.8)	0.77	(0.50, 1.20)	0.25
*High blood pressure*, n (%)*	221	(15.4)	62	(19.3)	1.31	(0.96, 1.79)	0.09
*Hours of sleep*, n (%)							0.06
≥ 8	270	(26.1)	52	(22.3)	1.00	(reference)	
6-7	675	(65.3)	150	(64.4)	1.15	(0.82, 1.63)	
≤ 5	88	(8.5)	31	(13.3)	1.83	(1.10, 3.03)	
*Smoking status*, n (%)							0.93
Never smoked	749	(51.9)	120	(51.8)	1.00	(reference)	
Ex-smoker	489	(33.9)	108	(33.1)	0.98	(0.75, 1.28)	
Current smoker	206	(14.3)	49	(15.0)	1.05	(0.74, 1.50)	

Note: *denotes categorical variables which have an omitted reference group of ‘no’.

Similarly, the pain and psychosocial characteristics of the sample together with the bivariable associations are presented in Table 2. The only factor that did not meet the inclusion threshold for the multivariable analysis was psychological demands. All remaining variables were considered in the multivariable model development.

**Table 2:** Pain and psychosocial characteristics of Australian and New Zealand nurses and midwives with neck pain in the preceding 12-months by whether they took no sick leave (n = 1511) or sick leave (n = 343), together with the bivariable associations between these factors and neck pain related sick leave (NP-SL)

	**No sick leave taken**		**Sick leave taken**		**OR**	**(95% CI)**	**p-value**
**Pain characteristics**							
*Neck pain severity*, mean (SD)	5.2	(2.2)	7.5	(1.8)	1.70	(1.59, 1.83)	<0.001
**Psychosocial factors: cognitive**							
*Fear of movement*, mean (SD)	20.8	(5.0)	24.2	(5.1)	1.15	(1.12, 1.18)	<0.001
*Pain catastrophising*, mean (SD)	6.4	(7.4)	8.4	(8.3)	1.03	(1.02, 1.05)	<0.001
Rumination subscale, mean (SD)	2.6	(3.0)	3.0	(3.2)	1.04	(1.00, 1.08)	0.03
Magnification subscale, mean (SD)	1.3	(1.7)	1.6	(1.8)	1.11	(1.04, 1.19)	0.001
Helplessness subscale, mean (SD)	2.5	(3.4)	3.7	(4.0)	1.08	(1.05, 1.12)	<0.001
*Belief work caused/exacerbated NP*, n (%)*	1232	(82.1)	312	(91.5)	2.35	(1.57, 3.51)	<0.001
**Psychosocial factors: behavioural**							
*Pain coping – passive coping*, mean (SD)	11.4	(3.6)	14.2	(4.0)	1.21	(1.17, 1.25)	<0.001
*Pain coping – active coping*, mean (SD)	16.7	(3.4)	16.5	(3.1)	0.98	(0.95, 1.02)	0.33
**Psychosocial factors: emotional**							
*Depressive symptoms*, n (%)*	347	(24.2)	118	(37.2)	1.86	(1.43, 2.40)	<0.001
*Diagnosed anxiety*, n (%)*	232	(16.2)	73	(22.7)	1.52	(1.13, 2.05)	0.01
*Diagnosed depression*, n (%)*	393	(27.4)	119	(37.1)	1.56	(1.21, 2.02)	0.001
*General mental health*, mean (SD)	46.1	(10.1)	42.8	(10.6)	0.97	(0.96, 0.98)	<0.001
**Psychosocial factors: social**							
*Skill discretion*, mean (SD)	37.6	(4.7)	37.1	(5.1)	0.98	(0.96, 1.00)	0.10
*Decision authority*, mean (SD)	36.0	(6.9)	35.4	(7.4)	0.99	(0.97, 1.01)	0.21
*Job control*, mean (SD)	73.6	(10.4)	72.6	(11.4)	0.99	(0.98, 1.00)	0.12
*Psychological demands*, mean (SD)	16.0	(3.1)	16.1	(3.2)	1.01	(0.98, 1.04)	0.62
*Co-worker support*, mean (SD)	6.0	(1.0)	5.9	(1.0)	0.92	(0.81, 1.03)	0.15
*Supervisor support*, mean (SD)	5.8	(1.5)	5.6	(1.7)	0.90	(0.84, 0.98)	0.01
*Job support*, mean (SD)	11.9	(2.2)	11.5	(2.4)	0.94	(0.89, 0.99)	0.02
*Over commitment*, n (%)							0.004
Low	729	(69.9)	142	(60.2)	1.00	(reference)	
High	314	(30.1)	94	(39.8)	1.54	(1.15, 2.06)	

Note: *denotes categorical variables which have an omitted reference group of ‘no’.

### Multivariable analysis

In developing the most parsimonious main effects logistic regression model, the sequentially eliminated variables (in order) were: active coping; manual handling task frequency; country; over commitment; hours of sleep; diagnosed depression; asthma; belief that nursing work caused/exacerbated NP; high blood pressure; marital status; job classification; physical demands; and depressive symptoms. Introducing the two-factor (age and gender) interaction terms and eliminating non-significant variables in a stepwise fashion yielded the adjusted odds ratio (aOR) estimates, together with the associated 95% CIs, presented in Table 3.

**Table 3:** Adjusted odds ratio (aOR) estimates and associated 95% CIs for the terms in the final multivariable logistic regression model

**Variables**	**aOR**	**(95% CI)**
Age^1^ (in years)	0.975	(0.959, 0.990)
General physical health score^2^	0.996	(0.975, 1.017)
Age^1^ × General physical health score^2^	1.002	(1.000, 1.004)
Neck pain severity score	1.593	(1.470, 1.727)
Passive coping score	1.079	(1.033, 1.126)
Fear of movement score	1.057	(1.022, 1.093)
General mental health score^2^	0.994	(0.979, 1.009)
Male^3^	0.876	(0.453, 1.695)
General mental health score^2^ × Male^3^	0.937	(0.890, 0.987)

^1^Centred around 45 years (median value); ^2^centred around 50 (population norm score); ^3^taking females as the reference category.

There were significant main effect relationships between NP-SL and neck pain severity, passive coping, and fear of movement. However, more complicated significant relationships emerged between NP-SL and age and general physical health scores, and between NP-SL and gender and general mental health scores; see Table 3. There was no reason to reject the adequacy of this final model (Hosmer-Lemeshow test, p = 0.96), and the model explained 31% of the NP-SL variance.

### Post hoc analysis

A *post hoc* analysis was performed to examine why pain catastrophizing was not retained in the final model. Pain catastrophizing was moderately correlated with passive coping (*R* = 0.39), and had modest correlations with the other significant variables: pain severity (*R* = 0.12); gender (*R* = -0.01); age (*R* = -0.11); general physical health score (*R* = -0.24); general mental health score (*R* = -0.25); and fear of movement (*R* = 0.24). Linear regression analysis of pain catastrophizing yielded tolerance estimates that ranged from 0.63 (passive coping) to 0.98 (age × general physical health score interaction) and a condition index estimate of 13.6, suggesting that multicollinearity among independent variable in not a significant issue.

In the bivariable analysis with NP-SL, pain catastrophizing was significant (p < 0.001) but explained only 2% of the variability of NP-SL. When a trivariable analysis was performed with passive coping added to the model, pain catastrophizing was no longer statistically significant (p = 0.26), and the model explained 14% of NP-SL variability. The interaction of pain catastrophizing and passive coping on NP-SL yielded an estimated coefficient of 0.0002 (Standard error [SE]: 0.0019, Wald’s χ^2^ statistic p = 0.91). These analyses suggest that the bivariable effect of pain catastrophizing on NP-SL was primarily confounded by passive coping.

## Discussion

The key objective of this study was to determine the differential role of pain-related cognitions and behaviours alongside other risk indicators in NP-SL. While many cases of sick leave due to incapacitating NP are likely to be appropriately exercised, fear-avoidance theory [15] suggests there may also be cases where fearful cognitions about pain prohibit work attendance rather than actual functional limitation. It is this NP-SL that has the potential for prevention if relevant psychological factors are identified and targeted interventions developed. In a cohort of nurses and midwives with NP in the preceding year, we found pain severity, fear of movement and passive pain coping increased the likelihood of work absence. In addition, there were complex interactions found between demographic and general health factors.

The relationship between neck pain severity and NP-SL was expected. Pain severity has consistently shown a relationship with NP-SL [43-45], and in our model, severity of worst pain in the preceding year increased the odds of NP-SL by 59% per unit increase in pain rating. Age and gender have also been significantly associated with NP-SL [45,46], although this study is the first to demonstrate significant effect modifications with general physical and mental health scores. This may partially explain some of the equivocal findings reported in earlier studies [46].

We found that fear of movement increased the odds of NP-SL in the preceding year by 6% per unit increase in TSK-11 score, suggesting that even after controlling for pain severity, nurses and midwives with NP who are fearful of movement are more likely to stay home from work. This is an exciting finding given that fear of movement has shown some potential for modification in an intervention study with individuals following back injury [47]. Further, the current finding is consistent with evidence from other studies that demonstrate fear of movement is related to sick leave due to low back pain (LBP-SL) [16,17]. In a previous analysis of LBP-SL in nurses and midwives, fear of movement increased the likelihood of LBP-SL by 5% for women and 8% for men [16]. Hence, the strength of association in this study is consistent with previous analyses for LBP-SL. In terms of Hill’s criteria for causation [48], there is mounting evidence for a causal relationship between fear of movement and sick leave due to spinal pain: the temporal relationship [17], strength and consistency of association in this and others studies [16,17], and plausibility of the relationship [15]. However, before causation can be asserted, all of Hill’s criteria need to be satisfied, including the yet to be established dose–response relationship between fear of movement and sick leave due to spinal pain, demonstrated prevention or amelioration by appropriate experimental regimen, and the establishment of coherency and adequate specificity.

Passive coping also increased the likelihood of NP-SL, by 8% per unit increase in score (on a 24-point scale). This finding adds weight to the importance of passive coping in musculoskeletal pain outcomes. Mercado and colleagues [49] demonstrated that individuals using moderate to high levels of passive coping strategies were five times more likely to develop disabling neck and/or low back pain than those with low levels. Passive coping has also been shown to increase the likelihood of LBP-SL by 7% per unit increase in score [16].

The significant interaction terms are illuminating. The first reveals that as age and general physical health scores together increase, the odds of NP-SL decrease less than if age and general physical health scores were considered separately as independent effects. Similarly, as age and general physical health scores together decrease, then the odds of NP-SL increase less than if considered separately. So, in workforce planning, both variables should be considered together rather than in isolation. The second interaction term reveals that the general mental health score association with NP-SL is primarily associated with males; and the lower the general mental health score a male has, the higher his odds of NP-SL. While significant here, interaction terms are rarely investigated.

Our analysis identified no direct relationship between pain catastrophizing and NP-SL. Our *a priori* hypothesis that pain catastrophising would be associated with NP-SL was based on fear-avoidance theory [14,15] which asserts pain catastrophizing leads to fear of movement and avoidant behaviours. While significant in the bivariable model, NP-SL was primarily confounded by passive coping in the multivariable model. This finding, and a similar non-significant relationship between pain catastrophising and LBP-SL [16], suggests that pain catastrophizing may be related to proximal factors in the fear-avoidance model but not directly related to work absence. Another rejected *a priori* hypothesis was that of an association between active coping and NP-SL. We found active coping had no relationship with NP-SL in either bivariable or multivariable analyses. A lack of association has also been demonstrated between active coping and NP-related disability [16,49]. According to fear-avoidance theory, active coping responses should represent confrontational behaviours that lead to recovery [14,15]. Given that flaws in the measurement of active coping have been proposed [49], further work is needed to establish valid and reliable measurement of active coping behaviours to facilitate further investigations into the possible protective influence of active coping on NP-SL.

Self-reported psychosocial work exposure such as job strain, low co-worker support, decreased job security and overall stress at work have also been shown to be risk factors for NP [45,46]. While many considered occupational and social variables had significant bivariable associations with NP-SL in this study, none appeared in the final multivariable model. This implies, for our study group, that demographic, general health, pain characteristics, and psychosocial factors mediated these occupational and social variables in explaining NP-SL. The relatively homogeneous and structured occupational and social characteristics around nursing and midwifery may hide the small but significant associations seen elsewhere [46].

An urgent call has been made for high quality randomised controlled trials of workplace interventions for neck pain [45]. Our findings advocate for multidimensional preventive interventions that incorporate strategies to ameliorate both fear of movement and passive coping behaviours. Fortunately, a coping-oriented cognitive and behavioural intervention developed by Linton and colleagues [50,51], is clearly defined and could readily be applied in future trials in nurses and midwives. However, that intervention did not reduce fear of movement [50,51]. Program strategies that target fear of movement, such as those included in the Pain-Disability Prevention Program [47], could be combined with the coping-oriented program in the hope of additive effects on NP-SL. Optimized pain management should be also considered in multidimensional NP-SL preventive interventions since pain severity is related to NP-SL and has also been shown to predict future fear of movement [52]. Given that pain severity, fear of movement and passive coping are related to both NP-SL (as describe here) and sick leave due to low back pain [16], a multidimensional program to address these factors is likely to have positive impacts on work loss due to both neck and low back pain.

This study has a number of strengths but also limitations. This is the first investigation of pain-related psychological factors and NP-SL and provides new insights into exposure relationships. Pain coping has been considered as a confounder in an analysis of sick leave due to a composite outcome of neck or upper extremity pain [12], but the main effect of pain-related psychological characteristics has never before been explored. There are three important limitations to consider: (i) the cross-sectional design precludes causal inferences; (ii) all NMeS data were self-reported and do not include objective measures; and, (iii) the NMeS sample represents less than 5% of the nursing and midwifery population that investigators attempted to invite [21,22].

Only with the following of NMeS participants over time, together with the acquisition of empirical information gleaned from other studies, will causal inferences able to be made. Self-report studies have validity problems; participants may exaggerate conditions or events in order to make their situation seem worse or through social desirability bias they might under-report conditions or events in order to conceal or minimize stigmatised matters. Participants may also simply be mistaken or misremember the material covered by the survey. However, our deliberate choice of standardised instruments sought to minimise this inherent effect.

The modest participation rate potentially threatens the external validity of the data, although the associated impact of differential participation in large-scale, multiple-domain, targeted population studies is likely to be small [22]. Selective participation in relation to NP is unlikely because potential participants were informed in NMeS promotional material about broad research objectives (i.e. to study workforce and health outcomes) that made no reference to NP. Moreover, in relation to basic demographic characteristics and NP prevalence, there is no obvious responder bias [21,22]. In this analysis, 50.6% of nurses and midwives who met inclusion criteria reported NP in the previous year. This is reassuringly consistent with a sample of New Zealand nurses with similar demographic characteristics who reported 52% prevalence using the same measurement instrument [53]. Further, in our sample 18.5% of nurses with NP reported NP-SL in the preceding year, compared to 21.3% of individuals in a large general population sample that reported neck, shoulder or upper back pain [54].

The overall NMeS registrants have been shown to be similar to the source Australian and New Zealand populations in relation to gender, age and Indigenous representation [21,22]. This similarity is notable as it might have been opined that older participants would have reduced access to, and have been less comfortable with, a fully electronic survey. However, the use of information technology is now considered a core aspect of modern practice and is a basic element in nursing and midwifery programmes [20]. Furthermore, Internet access at home is becoming commonplace. In 2008, 75% of Australian households had a computer and 67% had home Internet access, of which 78% was broadband [20]. Internet penetration is likely higher in our target populations as rates increase markedly with higher incomes. In respect to the sample of nurses and midwives included in this analysis, participants’ demographic characteristics (92.9% female and mean 43.9 years) were consistent with the Australian nursing and midwifery labour force at the time of recruitment (90.4% female and mean 43.7 years) [55]. Slight differences may reflect demographic associations with NP prevalence, since NP appears to differentially affect women (females report NP more than men in 83% of prevalence studies) [2].

## Conclusions

In summary, pain severity, fear of movement and passive coping behaviours are associated with sick leave due to NP in nurses and midwives. Other factors that relate to NP-SL include demographic and general health factors. Interestingly, occupational, social, and several cognitive factors (including pain catastrophizing) were not significantly associated with NP-SL in our sample. Our findings indicate that multidimensional interventions to reduce fearful cognitions about pain, ameliorate passive coping behaviours and optimise pain management should be developed and examined via high quality trials. Further research will determine whether such interventions can reduce the societal, workforce and individual costs of NP-SL.

## Abbreviations

aOR: Adjusted odds ratio; BIC: Bayesian information criterion; CESD-10: 10-item centre for epidemiolgic studies depression scale; CI: Confidence interval; ERI: Effort-reward imbalance; ERIQ: Effort-reward imbalance questionnaire; ICC: Intra-class correlation; JCQ: Job content questionnaire; LBP-SL: Sick leave due to low back pain; NP: Neck pain; NP-SL: Sick leave due to neck pain; NMeS: Nurses and midwives e-cohort study; OC: Over-commitment; SF-36v2: 36-item short form health survey version 2; TSK-11: 11-item Tampa scale of Kinesiophobia; UK: United Kingdom; US: United States; VPMI: Vanderbilt pain management inventory; SD: Standard deviation; SE: Standard error.

## Competing interests

The authors declare that they have no competing interests to declare.

## Authors’ contributions

PJS and APD conceived the idea for the paper. PJS performed the statistical analyses. PJS, APD and CT jointly drafted the manuscript with the main part of the introduction, methods and discussion written by APD and the main part of the results section drafted by PJS. All authors interpreted the data, reviewed iterations of the manuscript and approved the final version.

## Pre-publication history

The pre-publication history for this paper can be accessed here:

http://www.biomedcentral.com/1472-6955/13/5/prepub
